# Notch signaling and natural killer cell infiltration in tumor tissues underlie medulloblastoma prognosis

**DOI:** 10.1038/s41598-021-02651-y

**Published:** 2021-12-02

**Authors:** Kung-Hao Liang, Che-Chang Chang, Kuo-Sheng Wu, Alice L. Yu, Shian-Ying Sung, Yi-Yen Lee, Muh-Lii Liang, Hsin-Hung Chen, Jun-Jeng Fen, Meng-En Chao, Yi-Ting Liao, Tai-Tong Wong

**Affiliations:** 1 Department of Medical Research, Taipei Veterans General Hospital, Taipei, Taiwan; 2 Institute of Food Safety and Health Risk Assessment, National Yang-Ming Chiao-Tung University, Taipei, Taiwan; 3 Institute of Biomedical Informatics, National Yang-Ming Chiao-Tung University, Taipei, Taiwan; 4 The PhD Program for Translational Medicine, Taipei Medical University, Taipei, 110 Taiwan; 5 Graduate Institute of Clinical Medicine, College of Medicine, Taipei Medical University, Taipei, 110 Taiwan; 6 Institute of Stem Cell and Translational Cancer Research, Chang Gung Memorial Hospital at Linkou and Chang Gung University, Taoyuan, 333 Taiwan; 7 Genomics Research Center, Academia Sinica, Taipei, 115 Taiwan; 8 Division of Paediatric Neurosurgery, the Neurological Institute, Taipei Veterans General Hospital and School of Medicine, National Yang-Ming University, Taipei, 112 Taiwan; 9 Department of Informatics, Taipei Veterans General Hospital, Taipei, Taiwan; 10 Pediatric Brain Tumor Program, Taipei Cancer Center, Taipei Medical University, Taipei, 110 Taiwan; 11 Division of Pediatric Neurosurgery, Department of Neurosurgery, Taipei Medical University Hospital, Taipei Medical University, Taipei, 110 Taiwan; 12 Neuroscience Research Center, Taipei Medical University Hospital, Taipei, 110 Taiwan

**Keywords:** Molecular medicine, Cancer

## Abstract

Medulloblastoma is the most common embryonic brain tumor in children. We investigated a cohort of 52 Asian medulloblastoma patients aged between 0 and 19 years old, who received surgical resections and post-resection treatments in the Taipei Medical University Hospital and the Taipei Veterans General Hospital. Genome-wide RNA sequencing was performed on fresh-frozen surgical tissues. These data were analyzed using the CIBERSORTx immune deconvolution software. Two external clinical and molecular datasets from United States (n = 62) and Canada (n = 763) were used to evaluate the transferability of the gene-signature scores across ethnic populations. The abundance of 13 genes, including DLL1, are significantly associated with overall survival (All Cox regression P < 0.001). A gene-signature score was derived from the deep transcriptome, capable of indicating patients’ subsequent tumor recurrence (Hazard Ratio [HR] 1.645, confidence interval [CI] 1.337–2.025, P < 0.001) and mortality (HR 2.720, CI 1.798–4.112, P < 0.001). After the adjustment of baseline clinical factors, the score remains indicative of recurrence-free survival (HR 1.604, CI 1.292–1.992, P < 0.001) and overall survival (HR 2.781, CI 1.762–4.390, P < 0.001). Patients stratified by this score manifest not only distinct prognosis but also different molecular characteristics: Notch signaling ligands and receptors are comparatively overexpressed in patients with poorer prognosis, while tumor infiltrating natural killer cells are more abundant in patients with better prognosis. Additionally, immunohistochemical staining showed the DLL1 protein, a major ligand in the Notch signaling pathway, and the NCAM1 protein, a representative biomarker of natural killer cells, are present in the surgical tissues of patients of four molecular subgroups, WNT, SHH, Group 3 and Group 4. NCAM1 RNA level is also positively associated with the mutation burden in tumor (P = 0.023). The gene-signature score is validated successfully in the Canadian cohort (P = 0.009) as well as its three molecular subgroups (SHH, Group 3 and Group 4; P = 0.047, 0.018 and 0.040 respectively). In conclusion, pediatric medullablastoma patients can be stratified by gene-signature scores with distinct prognosis and molecular characteristics. Ligands and receptors of the Notch signaling pathway are overexpressed in the patient stratum with poorer prognosis. Tumor infiltrating natural killer cells are more abundant in the patient stratum with better prognosis.

## Introduction

Central nervous system tumors are the most common solid tumors in children^1,2^. Particularly, medulloblastoma, atypical teratoid/rhabdoid tumor (ATRT) and primitive neuroectodermal tumor (PNET) are three malignant embryonic tumors with the highest mortalities^2^. Medulloblastoma is most prevalent among these embryonic tumors, with an age-adjusted incidence of 0.20–0.58 cases per 100,000 children^2^. Current treatments of medulloblastoma comprise surgical resection and post-resection radiotherapy, with or without chemotherapy (in non-infants) or high dose chemotherapy and autologous stem cell rescue (in infants). The treatment outcome is still unsatisfactory due to the metastasis, recurrence, and the impairment of cognitive functions^3^.

In East Asia, the etiology, biology and the variability of post-treatment prognosis of medulloblastoma remain unclear. Biomarkers with strong associations with clinical endpoints, often reflected as large odds ratios or hazard ratios, may be used to guide personally tailored treatments^4^. MYC and MYCN are known proto-oncogenes^5,6^, while TP53 is a known tumor suppressor gene of medulloblastoma^7^. Somatic abnormality of MYC, MYCN and TP53 have been found to correlate with clinical outcome. Isochromosome 17q, a somatic DNA defect often accompanied by deletion of chromosome 17p which harbors TP53, are indicative of poor prognosis^8^. However, this DNA defect appears in ~ 30% of the patients^7,9,10^. Variability and heterogeneity of the disease is still seen in patients without these defects.

Investigations of gene signatures utilizing a collection of genes are warranted to characterize this disease more comprehensively. A general classification of medulloblastoma sub-diseases (WNT, SHH, Group 3 and Group 4) was accepted by medical communities and the world health organization^11,12^. However, substantial molecular heterogeneity and clinical variability still exist in each subgroup^13^. Therefore, more refined systems with more subgroups were offered subsequently, aiming to achieve more homogeneous molecular characteristics and clinical outcome in each subgroup. In a study conducted in Britain, 7 subgroups of medulloblastoma were proposed^14^. In a recent study in Canada, 12 subgroups were proposed^15^. These subgrouping systems require more biomarkers and more complex decision rules, limiting their practical use. A simple system with fewer numbers of subgroups and acceptable homogeneity remains an unmet medical need.

We recently reported a cohort of 52 medulloblastoma patients in the light of conventional molecular subgroups (i.e. WNT, SHH, Groups 3 and 4)^16^. To fully explore novel molecular insights underneath the clinical outcome, we further investigated a gene signature which can stratify patients with distinct prognosis. Tumor infiltrating immunological profiles were also investigated for elucidating their effects in patients’ prognosis.

## Results

### Derivation of a gene-signature risk score and identification of Notch activation in high-risk patients

**Figure 1:**
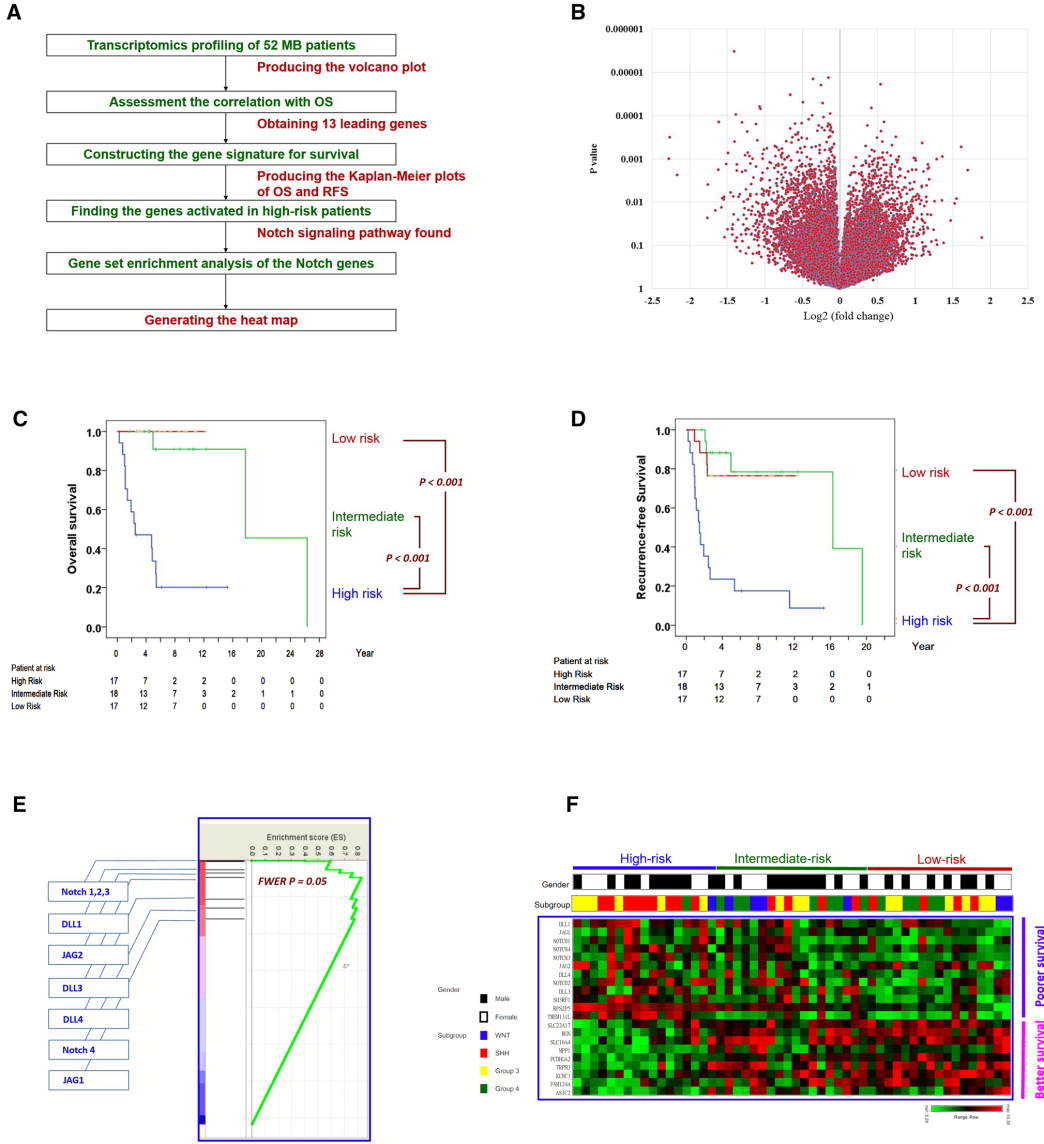
Derivation of a gene-signature based patient stratification score and identification of Notch activation in high-risk patients. (**A**) the flow chart; (**B**) the volcano plot of transcriptome-wise expression comparison of patients deceased and not deceased during the follow up; (**C**) the overall survival of patients stratified by the gene-signature based score; (**D**) the recurrence-free survival of patients stratified by the score. P values in the Kaplan–Meier plots were calculated by the log-rank test; (**E**) genes in the Notch pathway are at the leading edge of the rank of differentially expressed genes (the enrichment score = 0.83); (**F**) the heat map of gene levels in the 52 patients sorted by their gene-signature scores.

In this patient cohort, age and gender are not significantly associated with overall survival (Table 1). We explored gene signatures with respect to post resection clinical outcomes, in a pipeline summarized in Fig. 1A. A transcriptome-wide univariate analysis of gene levels in deceased and non-deceased patients was first performed, visualized as fold changes and statistical significance of each gene in the horizontal and the vertical axes respectively (Fig. 1B). Genes with large positive and negative log fold changes (> 1) and high statistical significance (< 0.01) were further investigated. The expression levels of these gene were then correlated with the patients’ overall survival using the Cox proportional hazards method. A total of 13 leading genes were found to have met three criteria simultaneously: (1) Cox P < 0.001, (2) T-test P < 0.001, (3) absolute (log2 (fold change)) > 1 (Table 1). A hazard ratio greater than 1 indicates that the gene is associated with poorer survival.

**Table 1:** A list of 13 leading genes significantly associated with overall survival.

Variables	Cox-regression analysis				
	HR	95% CI		P	
Gender male (%)	0.936	0.322	2.718	0.903	
Age at presentation (year)	0.946	0.827	1.082	0.415	
**Gene expression levels**					
ASIC2	0.565	0.365	0.874	**0.01**	Acid sensing ion channel subunit 2
DLL1	2.15	1.198	3.858	**0.01**	Delta like canonical notch ligand 1
FAM124A	0.246	0.107	0.565	**0.001**	Family with sequence similarity 124 member A
KCNC3	0.443	0.287	0.682	**< 0.001**	Potassium voltage-gated channel subfamily C member 3
MPP1	0.217	0.089	0.53	**0.001**	Membrane palmitoylated protein 1
PCDHGA2	0.338	0.189	0.604	**< 0.001**	Protocadherin gamma subfamily A, 2
RGN	0.398	0.243	0.651	**< 0.001**	Regucalcin
RPS2P5	4.093	2.08	8.056	**< 0.001**	Ribosomal protein S2 pseudogene 5
SH3RF1	1.966	1.249	3.096	**0.004**	SH3 domain containing ring finger 1
SLC16A4	0.31	0.173	0.556	**< 0.001**	Solute carrier family 16 member 4
SLC22A17	0.26	0.133	0.509	**< 0.001**	Solute carrier family 22 member 17
TMEM131L	1.921	1.303	2.832	**0.001**	Transmembrane 131 like
TRPM3	0.666	0.503	0.883	**0.005**	Transient receptor potential cation channel subfamily M member 3

Significant values are in bold.

The leading genes by the univariate analysis were then used for the derivation of a gene signature score with respect to patients’ survival. The score is formulated in the format of a multivariate Cox proportional hazards model. The hazard function is:

1 $$ {\text{H}}\left( {{\text{t}}|{\text{ R}}} \right) \, = {\text{ H}}_{0} \left( {\text{t}} \right){\text{ exp }}\left( {\text{R}} \right), $$

where the score R is defined as:

2 $$ \begin{aligned} {\text{R }} & = {\text{ ASIC2 }} \times \, \left( { - 0.{7}0{13}} \right) \, + {\text{ KCNC3 }} \times \, \left( { - 0.{6532}} \right) \, + {\text{ RPS2P5 }} \times \, \left( {{ 1}.{2271}} \right) \\ & \quad + {\text{ SLC22A17 }} \times \, \left( { - 0.{6753}} \right) \, + {\text{ TRPM3 }} \times \, \left( { \, 0.{2454}} \right). \\ \end{aligned} $$

This gene-signature score R comprise five leading genes in their alphabetical order (ASIC2, KCNC3, RPS2P5, SLC22A17 and TRPM3). The score reflects subsequent recurrence (Hazard Ratio [HR] 1.645, confidence interval [CI] 1.337–2.025, P < 0.001) as well as mortality (HR 2.720, CI 1.798–4.112, P < 0.001). Based on the score, patients were stratified into tertiles accordingly, each comprise 1/3 of all patients. The 10-year survival rates of the high-risk tertile, the intermediate-risk tertile and the low-risk tertile are ~ 20%, 90% and 100% respectively (Fig. 1C). Patients of the high-risk tertile have a significantly poorer overall survival than the other two tertiles (both P < 0.001, Fig. 1C) as well as poorer recurrence-free survival (both P < 0.001, Fig. 1D). Hence, we stratify patients into the high-risk stratum (i.e. the high-risk tertile, N = 17) and average-risk stratum (including the intermediate- and low-risk tertiles combined, N = 35) due to their distinct prognosis.

The leading gene, delta like canonical notch ligand 1 (DLL1), is a major ligand of the Notch signaling pathway, an oncogenic pathway which has been reported sporadically in the literature of medulloblastoma^17–22^ and neurodevelopment^23^ but has yet to be well established as a driving mechanism of medulloblastoma. We hypothesized that ligands and receptors in the Notch signaling pathway are comparatively abundant in patients of the high-risk stratum, which comprise mostly the SHH (N = 9, 52.94%) and Group 3 (N = 6, 35.29%) patients (Table 2). To evaluate thus, we performed the Gene Set Enrichment Analysis (GSEA)^24^ of the ligands (DLL1, DLL3, DLL4, Jag1 and 2) and the receptors (Notch 1, 2, 3 and 4). All these genes turned out to locate at the leading edge of the list of all human genes, ranked by their differential expressions between patients of with poorer prognosis and those with better prognosis (Fig. 1E, family-wise error rate P = 0.05). Figure 1F presents the heat-map of the gene levels of patients sorted by their gene-signature score.

**Table 2:** Patient distributions in gene-signature risk subgroups and the conventional molecular subgrouping.

	Conventional molecular subgrouping				Sum
	WNT	SHH	Group 3	Group 4	
**Gene signature**					
High risk	1	9	6	1	17
Intermediate risk	4	4	3	7	18
Low risk	2	4	6	5	17
Sum	7	17	15	13	52

### Evaluation of gene-signature score performances in various clinical subgroups

**Figure 2:**
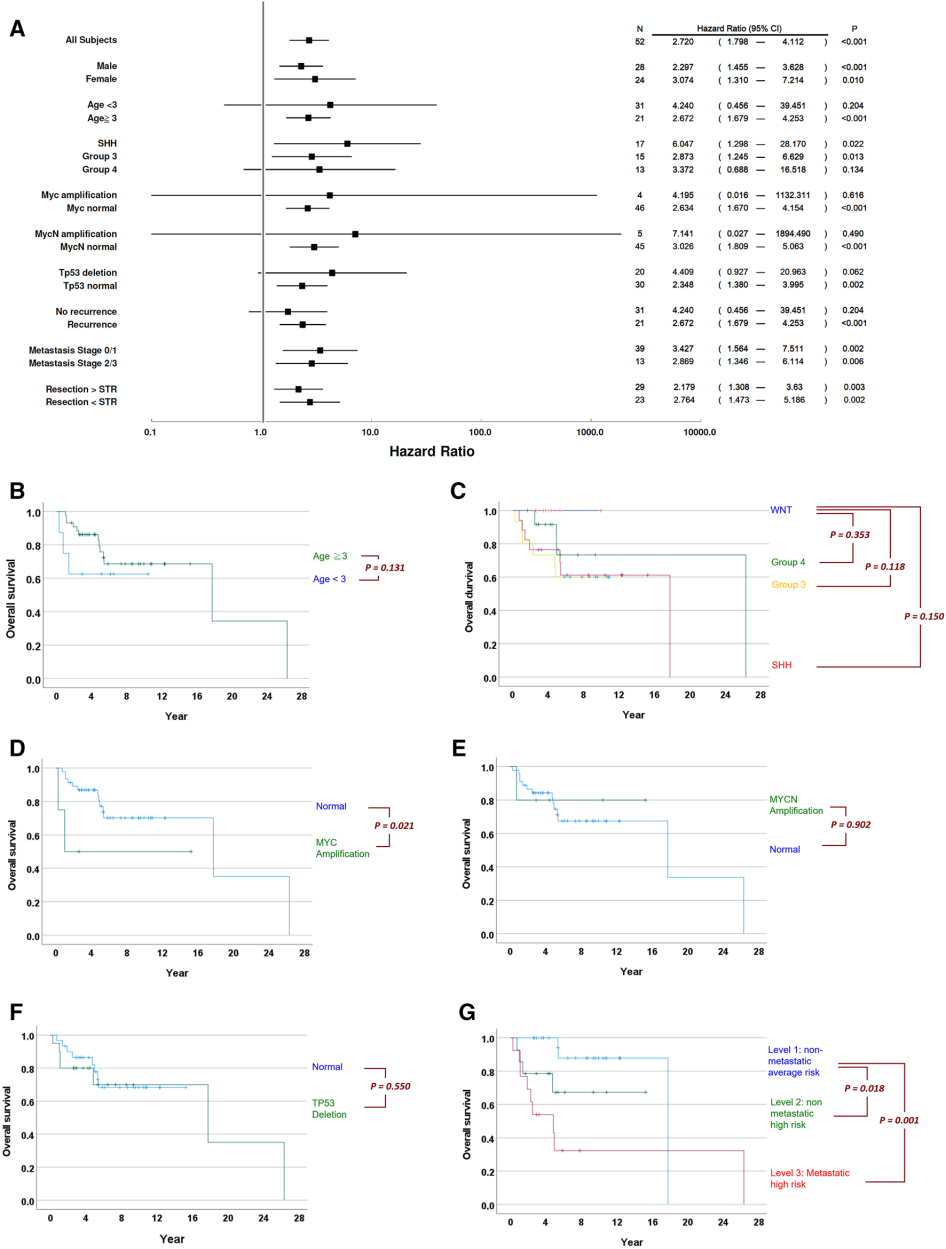
Evaluation of gene-signature score performances in various clinical subgroups. (**A**) The forest plot of the overall survival hazard ratios with respect to the gene-signature derived score in various clinical subgroups. Horizontal bars indicate the 95% confidence intervals of the hazard ratios. (**B**) The overall survival of patients stratified by age < 3 and ≧ 3. (**C**) The overall survival of patients stratified by molecular subgroups. (**D**) The overall survival of patients stratified with respect to somatic MYC amplification. (**E**) The overall survival of patients stratified with respect to somatic MYCN amplification. (**F**) The overall survival of patients stratified with respect to somatic TP53 deletion. (**G**) The overall survival of patients stratified by the clinical risk stratification scheme. P values in the Kaplan–Meier plots were calculated by the Breslow test.

We further evaluated whether the gene-signature score can indicate patients’ prognosis in specific patient subgroups, for example, in male and female patients**.** The score correlates significantly with overall survival in both genders (P < 0.001 and = 0.010 respectively, Fig. 2A). This analysis can also reveal the added value of this score given the currently known prognosis indicators, such as age, molecular subgroups^11,12^, somatic MYC/MYCN amplifications and TP53 deletions^7^. Patients younger than 3 years old have inferior overall survival (Fig. 2B). The score correlates significantly with patients ≧ 3 years old but not with patients < 3 years old (Fig. 2A). Patients in the WNT subgroup have better survival than those in SHH, group 3 and group 4 (Fig. 2C). The gene-signature score can further indicate overall survival of patients in SHH and group 3, but not in group 4 (Fig. 2A). Patients in the WNT subgroup all survived till the end of study, thus the Cox regression analysis cannot be performed.

Somatic DNA methylation status of 50 patients in our cohort (96.2%) have been examined using methylation arrays^16^. The sliding–window analysis of fluorescent intensities detected by the arrays offer information regarding somatic DNA amplifications and deletions^25^. Patient with the MYC amplification have inferior overall survival than those without this mutation (Breslow P = 0.021, Fig. 2D). Patient stratified by MYCN amplifications and TP53 deletions, on the other hand, does not manifest significant difference in terms of overall survival (Fig. 2E,F). The gene-signature score significantly correlates with clinical outcome in MYC normal, MYCN normal or TP53 normal patients (Fig. 2A). The gene-signature score sensitively correlates with mortality in patients with subsequent recurrence (P < 0.001), yet it is insensitive in patients without recurrence (P = 0.185). The score is effective in patients at either “metastasis stage 0/1” (P = 0.002) or “metastasis stage 2/3” (P = 0.006). The score is also predictive of prognosis in patients treated with > subtotal resection (STR) (P = 0.003) and patients with ≤ STR (P = 0.002).

We then evaluated the independence of the gene signature-based score and a simple clinical risk stratification scheme proposed previously, where the patients were stratified into three groups, non-metastatic average risk (N = 25), non-metastatic high risk (N = 14), metastatic high risk (N = 13)^16^. Both the non-metastatic and metastatic high risk subgroups have significantly poorer overall survival than the non-metastatic average risk group (Fig. 2G). The genetic signature and the clinical risk stratification scheme independently correlate with overall survival (P < 0.001 and = 0.033 respectively, Table 3) as well as recurrence-free survival (P < 0.001 and = 0.007 respectively, Table 3). The adjusted hazard ratios of the score are 2.781 (CI 1.762–4.390) for overall survival and 1.604 (CI 1.292–1.992) for recurrence-free survival.

**Table 3:** Univariate and multivariate analysis of the clinical risk stratifications and the gene-signature based score using Cox regressions.

Overall survival	Univariate analysis				Multivariate analysis			
	HR	95% CI		P	HR	95% CI		P
Clinical risk stratifications	2.621	1.392	4.936	0.003	2.344	1.069	5.141	0.033
The gene signature	2.720	1.798	4.112	< 0.001	\\2.781	1.762	4.390	< 0.001
**Recurrence-free survival**								
Clinical risk stratifications	2.349	1.434	3.849	0.001	2.098	1.220	3.608	0.007
The gene signature	1.645	1.337	2.025	< 0.001	1.604	1.292	1.992	< 0.001

The Heidelberg molecular and outcome-based scheme is a hybrid scheme combining known molecular and clinical factors^26^. An adjusted version was proposed based on patient data in Taiwan^16^. Patient stratifications based on the gene-signature and the adjusted scheme are shown in Supplementary Table 1. Two patients are unable to be classified by the adjusted scheme. For the rest of the patients, 30% were ascribed to have very-high and high risks; a majority of patients (54%) were ascribed to standard risks, while 16% were ascribed to low risk. The score and Heidelberg molecular and outcome-based scheme can stratify patients independently (Chi-square test, P = 0.121, N = 50).

### Tumor infiltrating immune cells inferred from the de-convoluted transcriptome and confirmed by immunohistochemical staining.

**Figure 3:**
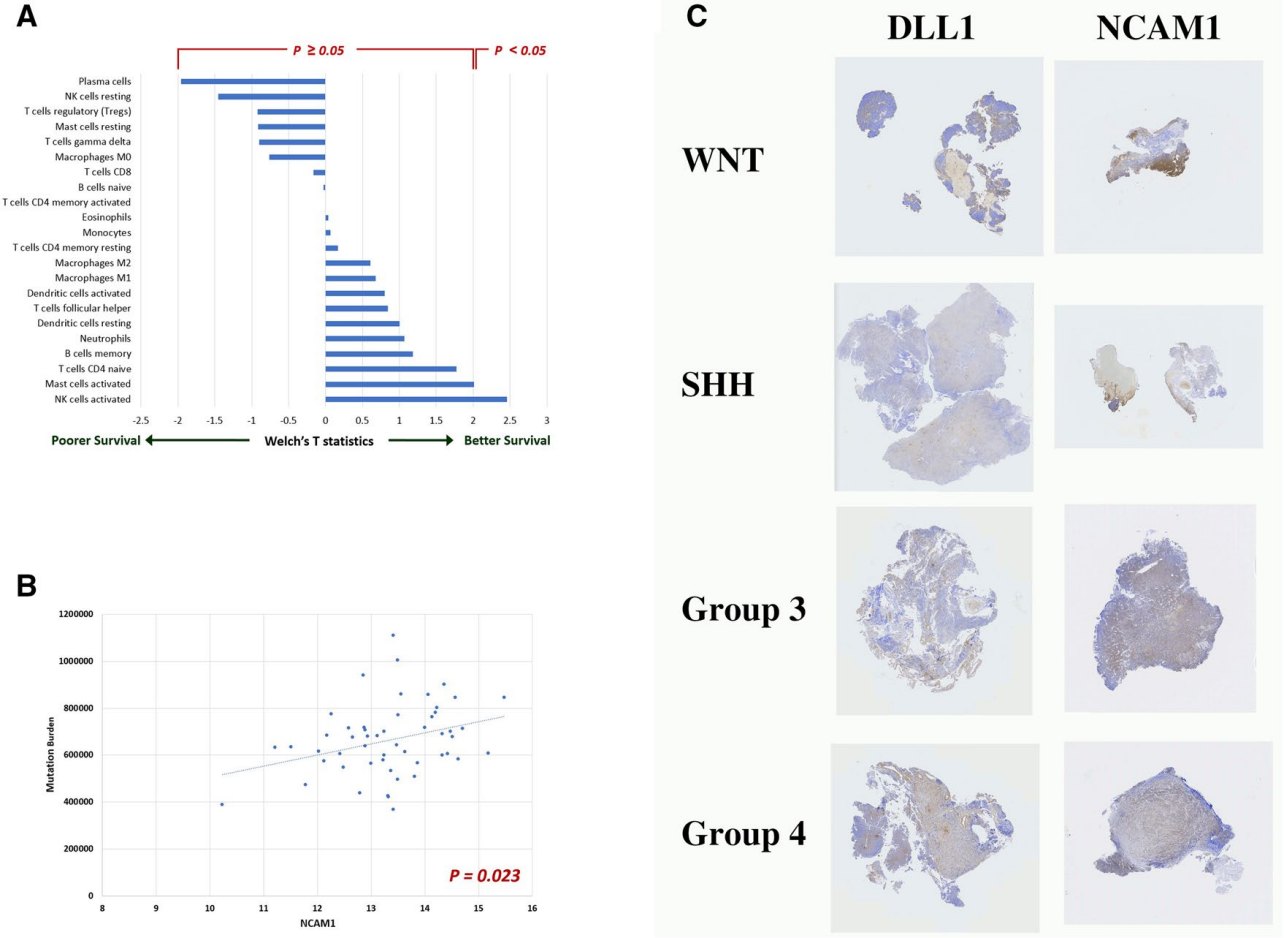
Tumor infiltrating immune cells inferred from the de-convoluted transcriptome and confirmed by immunohistochemical staining. (**A**) A comparison of 22 types of tumor infiltrating immune cells between patients with distinct prognosis. The natural killer (NK) cells manifest significantly higher quantity in patients with better prognosis (P = 0.020). The mast cells manifest borderline significance (P = 0.050). (**B**) The linear relationship between the NCAM1 (*a.k.a.* CD56) level, a representative biomarker of NK, and the somatic mutation burden (P = 0.023). (**C**) The immunohistochemistry staining of DLL1 and NCAM1 in tumor tissues of 8 medulloblastoma patients. The detected DLL1 and NCAM1 proteins are shown as brown color in the images.

A total of 22 tumor-infiltrating immunological cell types were inferred from the deep transcriptome using the CIBERSORTx immune deconvolution software, a well-known data-driven tool for cancer biology^27–29^. We compared the quantities of these immune cells in patient strata with poorer prognosis (high risk stratum) and better prognosis (average risk stratum). The tumor-infiltrating Natural Killer (NK) cells were found to have significantly higher quantity in patients with better prognosis (P = 0.020, Fig. 3A. The mast cells were found to manifest borderline significance (P = 0.050). The other cell types does not manifest significant difference (Fig. 3A).

The mutation burden was also found to be higher in patients with the better prognosis (P = 0.002). Interestingly, one representative biomarker of natural killer cells, NCAM1 (*a.k.a.* CD56), manifests a positive correlation with the mutation burden (P = 0.032, Fig. 3B). Hence, patients with better prognosis have more infiltrating natural killer cells in tumor, probably due to the neoantigens caused by the mutations, which are recognizable by the human immune systems.

We then performed the immunohistochemical (IHC) staining of DLL1, a representative protein in the Notch signaling pathway, and NCAM1, a representative protein of infiltrating natural killer cells, in the tumor tissues of 8 patients (Fig. 3C). Both proteins of DLL1 and NCAM1 are detected in medulloblastoma tissues of patients in four subgroups: WNT, SHH, group 3 and group 4.

### Transferability of gene signatures across datasets

**Figure 4:**
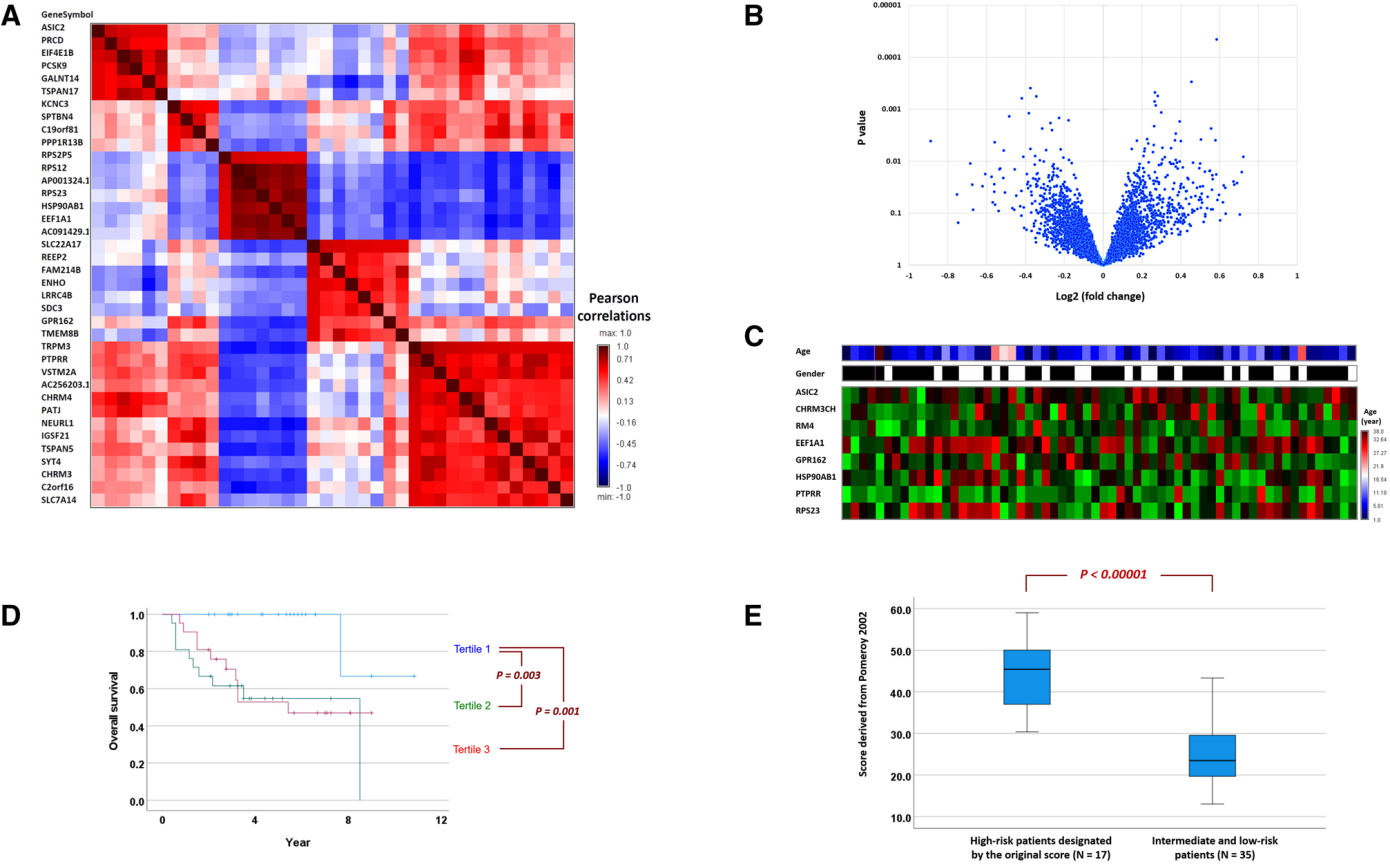
Transferability of gene signatures across datasets. (**A**) Pairwise correlations of a set of 38 genes which are highly correlated with one of the five leading genes, ASIC2, KCNC3, RPS2P5, SLC22A17 and TRPM3 (Pearson’s correlation > 0.65), and are referred to as the core-transferability gene set. Highly correlated genes are the surrogates of each other for the transfer of models across datasets. (**B**) The volcano plot of the transcriptome-wide comparisons of patients of the United States cohort who have or have not remained alive during the follow-up. (**C**) Age, gender and gene expressions of 8 genes of the United States cohort. All the 8 genes are within the core-transferability gene set. (**D**) Patient strata by the gene-signature score generated from the 8 genes manifest distinct overall survival. P values in the Kaplan–Meier plots were calculated by the log-rank test; (**E**) The high-risk stratum and average-risk stratum of the Taiwan cohort manifest distinct gene-signature score derived from the Pomeroy dataset.

Genes with mutual correlations in expression levels are useful for the transferability of gene signature across datasets. Should some genes are missing in other datasets, the correlated surrogate genes may be of use. A set of 38 genes was found to have high correlations to one of the five major genes in the gene signature defined by Eq. (2), ASIC2, KCNC3, RPS2P5, SLC22A17 and TRPM3 (All Pearson’s correlation > 0.65, Fig. 4A), and is referred to as the core-transferability gene set.

The ArrayExpress E-GEOD-68956 dataset is an iconic public-domain resource pertaining to medulloblastoma patients in United States. This is the first dataset clearly indicating medulloblastoma as molecularly distinct tumors from other embryonic brain tumors such as PNETs^30^. It is deposited to two large public-domain archives, the Gene Omnibus (GSE68956) and the ArrayExpress (E-GEOD-68956). Additionally, it was used for the demonstration of gene expression data analysis using the Matlab programming language in the MathWorks website. We employed this external dataset to evaluate the transferability of gene signatures across ethnic groups. Patients in this external dataset have long periods of follow up (mean ± standard deviation: 50.4 ± 30.2 months). A majority of the patients are male (66.1%). The age distribution is 8.1 ± 7.0 years. The tumor-tissue transcriptome were quantified by use of 7129 probe sets in microarrays. For the sake of visual quality inspection, a volcano plot was generated for the transcriptome (Fig. 4B), where the data distribution is well balanced without obvious skew or defect.

The microarray used for generating this dataset is Affymetrix Human Full Length HuGeneFL Array [Hu6800], an early design which did not contain all known human genes today. To transfer our gene signature into the external dataset, we employed the core-transferability gene set in Fig. 4A. Among the 38 surrogate genes, 8 genes were found in the external dataset (Fig. 4C). A transferred score was derived using these genes:

3 $$ {\text{Score }} = {\text{ ASIC2 }} \times \, \left( { - {1}.{2734}} \right) \, + {\text{ CHRM3 }} \times \, \left( { - {4}.{8683}} \right) \, + {\text{ CHRM4 }} \times \, \left( { - 0.{23}0{1}} \right) \, + {\text{ EEF1A1 }} \times \, \left( { - {1}.{8187}} \right) \, + {\text{ GPR162 }} \times \, \left( { - {1}.{442}0} \right) \, + {\text{ HSP9}}0{\text{AB1 }} \times \, \left( {{ 9}.{7673}} \right) \, + {\text{ PTPRR }} \times \left( {{ 1}.{4}0{14}} \right) \, + {\text{ RPS23 }} \times \, \left( { - {1}.{35}0{9}} \right). $$

The transferred score is correlated with the overall survival in this external dataset (HR 1.092, CI 1.014–1.176, P = 0.020). When patients were stratified into tertiles, stratum 1 has a favorable outcome compared with strata 2 and 3 (P = 0.003 and 0.001 respectively, Fig. 4D).

The transferred score was then applied to the gene expression levels of our patients in Taiwan. The gene-signature score and the transferred score are highly correlated with each other (Pearsaon’s correlation = 0.670, P < 0.001). The value distributions of the transferred score differ significantly in the high-risk and average-risk strata by the original score (Fig. 4E, P < 0.00001). Expression levels of the 8 genes in the Pomeroy dataset (N = 62) and in our Asian cohort (N = 52) are listed in the Supplementary Tables 2 and 3.

### Validation of gene signatures in a large Canadian cohort

**Figure 5:**
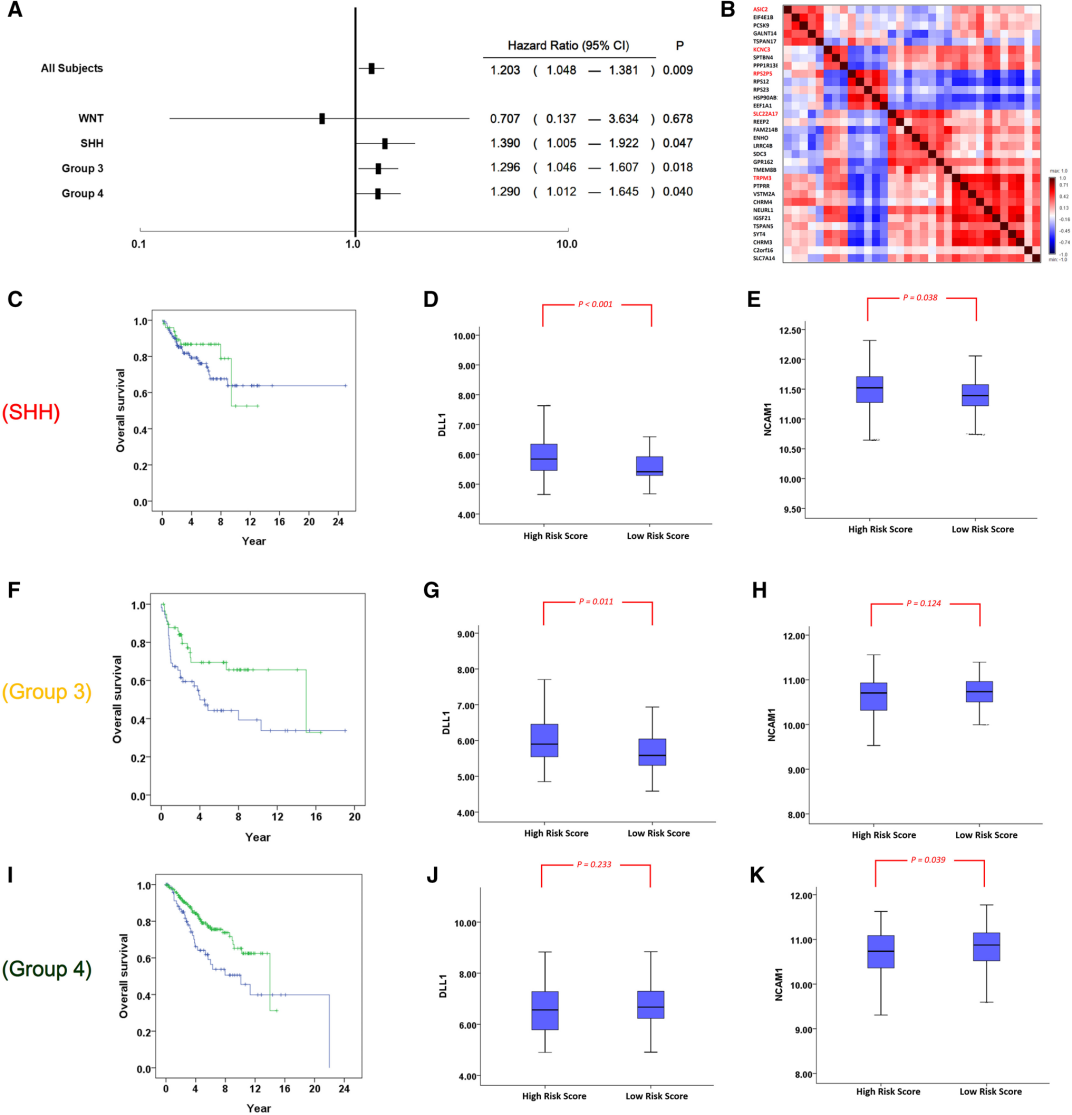
Validation of gene signatures using a large public-domain dataset. (**A**) The forest plot of the gene signature score performance in indicating overall survival of patients in the Canadian cohort and in the molecular subgroups. The score is significantly associated with overall survival in SHH (P = 0.047), Group3 (P = 0.018) and Group 4 (P = 0.040) patients. (**B**) Pairwise correlations of the levels of genes in the core-transferability gene set. (**C**–**E**) The overall survival, the distributions of DLL1 and NCAM1 genes of SHH patients stratified by median score into high-risk (shown as the blue curve in **C**) and low-risk (shown as the green curve in **C**) patients. (**F**–**H**) The overall survival, DLL1 and NCAM2 distributions of patient strata of Group 3. (**I**–**K**) The overall survival, DLL1 and NCAM2 distributions of patient strata of Group 4.

We validated the gene-signature score, derived from the Asian cohort and defined in Eq. (2), using data of a large Canadian cohort^15^. The cohort comprise 763 medulloblastoma patients. Among them, 612 patients have follow-up information which can be used for survival analysis. This dataset comprise gene expressions in fresh-frozen surgical samples, which have been measured using the Affymetrix Human Gene 1.1 ST Array platform. This is a relatively new microarray that the five major genes of the gene signature, ASIC2, KCNC3, RPS2P5, SLC22A17 and TRPM3, have all been assessed. Hence, we calculated the gene signature score of the Canadian patients using Eq. (2) without modifications. The score is significantly associated with overall survival (HR 1.293, CI 1.048–1.381, P = 0.009, Fig. 5A). There are 63, 172, 113 and 264 patients in the WNT, SHH, Group 3 and Group 4 with the follow-up information. The gene signature score is significantly associated with overall survival of the SHH, Group 3 and Group 4 patients in this cohort (P = 0.047, 0.018 and 0.040 respectively, Fig. 5A). Correlations of gene levels in the core-transferability gene set of the Canadian cohort are shown in Fig. 5B, which is visually similar to Fig. 4A. Patients in the Canadian cohort were then stratified by the median score values into high-risk and low-risk patients. Distinct overall survival of patient strata were visualized using the Kaplan–Meier plots in Figs. 5C (for SHH patients), 5F (for Group 3) and 5I (for Group 4), as the gene signature scores in these subgroups have been found to be significantly associated with overall survival (Fig. 5A). The gene level distributions of DLL1 (Fig. 5D,G,J) and NCAM1 (Fig. 5E,H,K) are also presented. It was found that the DLL1 levels were significantly higher in the high-risk patient stratum than low-risk patient stratum in SHH and Group 3 patients (P < 0.001 and = 0.011 respectively, Fig. 5D,G). In the meantime, the NCAM1 levels were significantly higher in the low-risk patient stratum than high-risk patient stratum in Group 4 patients (P = 0.039, Fig. 5K).

## Discussion

Investigations of cancer subgroups were performed conventionally by the following steps: (1) grouping co-expressed genes into molecular subgroups; (2) assigning patients into subgroups according to their molecular characteristics; (3) analyzing potential cancer-driving mechanisms (e.g. WNT, SHH) for the subgroups, and (4) investigating the average prognosis in each subgroup. We have recently reported our clinical experiences and outcomes of medullablastoma patients in Taiwan based on molecular subgroups defined using the above approach^16^. In the current study, we employed a different approach, i.e. finding a gene signature which can stratify patients with distinct prognosis, then analyzing the salient molecular characteristics of the patient strata. This different approach produced new insights. We discovered that in patients with poorer prognosis, ligands and receptors in the Notch signaling pathway are overexpressed. The protein of the DLL1 gene, one of the leading genes in our initial univariate analysis, was detected by IHC in our medulloblastoma tissues. The Notch signaling pathway, alongside other oncogenic pathways such as the WNT and SHH, have been considered as therapeutic targets of medulloblastoma^22^. A recent study showed the suppression of the Notch pathway can reduce medulloblastoma cell invasion, migration and angiogenesis^31^. Notch1 has been shown to induce SHH medulloblastoma^32^ and regulate the initiation of metastasis and self-renewal of Group 3 medulloblastoma in animal studies^21^. The high-risk stratum with Notch activation comprise mostly the SHH (N = 9, 52.94%) and Group 3 (N = 6, 35.29%) patients (Table 2). In our Asian data, 52.94% patients in the SHH group and 40.00% in Group 3 have poorer prognosis who also have Notch overexpression in tumor tissues (Table 2). This implies that subsets of SHH and group 3 patients have molecular characteristics of Notch overexpression, and these patients have particularly poor prognosis.

On the other hand, the tumor infiltrating natural killer cells were found to be more abundant in patients with better prognosis. Natural killer cells have been reported to deter or stabilize medulloblastoma in either cell based assays or early-phase clinical trials^33–35^, a possible explanation why these patients have better prognosis. Immunological cells have been observed to be able to infiltrate murine brains^36^. The NCAM1 protein, a representative biomarker of NK cells, was detected by IHC in the surgical tissues. Our data also showed that the NCAM1 gene level is linearly correlated with mutation burden, i.e. the number of mutations found in the expressed genes in tumor. The relationship between immune cells and somatic mutation load has been reported^37^. Currently, immunotherapy such as PD-L1, PD-1 antagonists and Chimeric antigen receptor T cell (CAR-T) are heavily investigated in multiple cancers including medulloblastoma. All the immunotherapies are based on the hypothesis that tumors harbor neoantigens recognizable by the human immunological system^37^. A higher mutation burden may result in more recognizable neoantigens, which is in agreement with our data.

Apart from molecular subgroups, we also evaluated the gene signature in other known subgroups, such as age < 3 and≧ 3 (Fig. 2A), with or without somatic MYC amplification, with or without MYCN amplification, and with or without TP53 deletion. Data suggested that when the patients with these known risk factors were excluded, our gene signature can still stratify patients with distinct outcome (Fig. 2A).

The current study is a retrospective study limited by the sample size of the Asian cohort. Compared with other common solid tumors such as non-small-cell lung cancer or hepatocellular carcinoma, the incidence of medulloblastoma is relatively low. Hence, patient enrollment requires much longer time than other studies. To cope with this limitation, we demonstrated the transferability of the gene signature into two public-domain datasets. Particularly, the Canadian cohort comprise a total of 763 medulloblastoma patients which is a decent sample size. Using this large dataset, we were able to show that the gene signature score derived from our Asian cohort is indicative of overall survival not only in SHH and Group 3 subgroups (Fig. 5A), echoing our previous finding in Fig. 2A, but also in Group 4 patients of the Canadian cohort. Furthermore, the DLL1 levels are significantly higher in the high-risk patient strata than the low-risk patient strata in SHH and Group 3 patients, while the NCAM1 levels are significantly higher in the low-risk patient stratum than high-risk stratum in Group 4 patients (Fig. 5). Despite the validation study, a prospective study would be important for revealing the predictability of the gene signature, which will be our future research direction.

In conclusion, we observed that the Notch signaling and infiltrating natural killer cells underlie the prognosis of medulloblastoma patients in Taiwan. The Notch ligands and receptors are overexpressed in the patient subgroup with comparatively poorer prognosis. The infiltration of natural killer cells in the tumor microenvironment is observed in the patient subgroup with better prognosis. These molecular characteristics of the patients shed light on future treatment strategies.

## Methods

### Patients

This is a retrospective study approved by the institutional review board of the Taipei Medical University Hospital and Chang Gung Memorial Hospital, and was performed in accordance of the Declaration of Helsinki ethical principles on human studies. A cohort of 52 medulloblastoma patients were investigated. These patients were treated by surgical resections in the Taipei Medical University Hospital (TMUH) and the Taipei Veterans General Hospital (TVGH). The age at presentation is between 0 and 19 years old (7.21 ± 4.15). Among them, 28 (53.85%) are male. All patients have given informed consent by themselves or by parent or legal guardian, if participants are under 18. Patients were followed until 2019/3/15 (the end of the study).

### Next generation RNA sequencing

The genome-wide RNA profiling (designated as RNAseq) was performed using the next generation sequencing technology, performed in a Nextseq 500 sequencing instrument (Illumina, San Diego, CA, USA) for multiplexed paired-end reads. Gene expression levels were quantified from the RNAseq data using Kallisto^38^ and tximport^39^ packages of the R statistical software. Mutation burdens were calculated using a pipeline comprising the Burrows-Wheeler Aligner (BWA), Samtools and bcftools. Tumor infiltrating immune cell profiles were also inferred from the transcriptome using the CIBERSORTx immune deconvolution algorithm and with the LM22 immune gene signature^27–29^.

### Evaluation of somatic DNA amplification and deletions

Somatic DNA amplifications and deletions were inferred from the intensity values detected by the methylation array, which is similar to a single nucleotide polymorphism (SNP) array in its technology.

### Immunohistochemistry staining of DLL1 and NCAM1 in resected HCC tissues

Immunohistochemistry staining of DLL1 and NCAM1 was performed on surgically resected medulloblastoma tissues preserved in the formalin-fixed paraffin-embedded (FFPE) blocks and cut into sections with a thickness of 5 μm. The FFPE samples were deparaffinized, dehydrated and incubated with the antibodies of DLL1 (ab84620, Abcam, Cambridge, UK) and NCAM1 (#3576S, Cell signaling technology, Danvers, MA, USA) for the staining.

### Processing of the dataset from United States

We extracted the clinical data of 62 medulloblastoma patients from the public-domain resource ArrayExpress (E-GEOD-68956) and then double checked with the supplementary table of the major reference paper Peromey et al*.* 2002^30^. The array design CDF file of Affymetrix GeneChip HuGeneFL Array [HuGeneFL] was also downloaded from ArrayExpress website. We then downloaded the raw intensity (CEL) files containing the tumor-tissue transcriptome of 62 medulloblastoma patients from the Gene Expression Omnibus (GSE68956). The CDF and CEL files were loaded into the RMAexpress software, where the gene expression levels were log2-transformed, background adjusted, quantile normalized, median-polish summarized using standard parameters, and then exported^40–42^. This data were then used for producing the volcano plot for visual inspections. Filtered by the core-transferability gene set, the expression levels of 8 genes were then used for the derivation of a translated score. The score was then applied to the gene expression levels of patients in the Asian cohort to check the value distributions in our patient strata with distinct clinical outcomes.

### Processing of the dataset from Canada

The major reference of this Canadian dataset is Calvalli et al*.*^15^. The normalized and log-transformed gene expression levels of 763 medulloblastoma patients, quantified by the Affymetrix Human Gene 1.1 ST Array platform, were downloaded from Gene Expression Omnibus archive (ID: GSE85217). The clinical data were downloaded from the from the website (https://ars.els-cdn.com/content/image/1-s2.0-S1535610817302015-mmc2.xlsx)^15^, which contained the censored/non-censored time-to-death information of 612 patients. The gene expression levels and the clinical data were joined for survival analysis.

### Statistical analysis

Survival analysis was performed using the Cox proportional hazards model. Cumulative incidence of death of different patient strata were compared using log-rank tests and visualized using Kaplan–Meier plots. Gene levels and tumor infiltrating cell quantity were compared using Welch’s t test. The IBM SPSS software version 20 (IBM, Armonk, NY) was used. The score were constructed by the multivariate combination of variables using the Generalized Iterative Modeling algorithm, which are capable of finding the optimum combination of genes with respect to various clinical criteria, in this case, the likelihood function as in the Cox regression^43,44^. The software code can be downloaded freely at the public-domain Github website (https://github.com/khliang/GIM).

## Supplementary Information

Supplementary Information.
