# Supplementary material for: Notch signaling and natural killer cell infiltration in tumor tissues underlie medulloblastoma prognosis
Source: Sci Rep. 2021 Dec 2;11:23282. doi: 10.1038/s41598-021-02651-y (PMC8639846; doi:10.1038/s41598-021-02651-y)
Supplement: Supplementary file 1 — Supplementary Information. [file 41598_2021_2651_MOESM1_ESM.docx]

**Notch signaling and Natural Killer cell infiltration in tumor tissues underlie medulloblastoma prognosis**

Kung-Hao Liang^1,2,3,*,#^, Che-Chang Chang^4,#^, Kuo-Sheng Wu^5^, Alice L. Yu^6,7^, Shian-Ying Sung^4^**,** Yi-Yen Lee^8^, Muh-Lii Liang^8^, Hsin-Hung Chen^8^, Jun-Jeng Fen^9^, Meng-En Chao^10^, Yi-Ting Liao^1^ and Tai-Tong Wong^10,11,12,13*^

**Supplementary Table 1**. Patient strata by the gene-signature risk score and the adjusted Heidelberg molecular and outcome-based risk stratification.

|  |  | Adjusted Heidelberg molecular and outcome-based risk stratification | | | | |  |
| --- | --- | --- | --- | --- | --- | --- | --- |
|  |  | Low risk | Standard risk | High risk | Very high risk | Unclassified | Sum |
| Gene signature | High risk | 1 | 6 | 3 | 5 | 2 | 17 |
|  | Intermediate risk | 5 | 9 | 3 | 1 | 0 | 18 |
|  | Low risk | 2 | 12 | 2 | 1 | 0 | 17 |
|  | Sum | 8 | 27 | 8 | 7 | 2 | 52 |

**Supplementary Table 2.** The level of co-transferability gene sets and the overall survival events and time (in months) of the United States cohort. The Translated Score was also calculated.

| OSEvent | OSTime | Array | ASIC2 | CHRM3 | CHRM4 | EEF1A1 | GPR162 | HSP90AB1 | PTPRR | RPS23 | Translated Score |
| --- | --- | --- | --- | --- | --- | --- | --- | --- | --- | --- | --- |
| 1 | 11 | GSM1688666_MG1999060202AA.CEL | 9.029462 | 6.70581 | 10.11065 | 13.75182 | 8.319473 | 12.43455 | 5.966329 | 11.48346 | 30.82263 |
| 1 | 5 | GSM1688667_MG2000030311AA.CEL | 9.194898 | 7.456076 | 9.974055 | 11.49004 | 8.662795 | 11.857 | 6.65063 | 13.26556 | 23.51968 |
| 1 | 7 | GSM1688668_MG1999052812AA.CEL | 9.486573 | 7.439467 | 10.1233 | 12.82856 | 9.04196 | 11.86582 | 6.663184 | 11.95771 | 22.08418 |
| 1 | 7 | GSM1688669_MG1999052811AA.CEL | 9.844251 | 6.879615 | 10.62352 | 12.81984 | 8.874406 | 11.98149 | 6.243382 | 11.75684 | 25.30948 |
| 1 | 7 | GSM1688670_MG1999062405AA.CEL | 9.639063 | 6.671095 | 9.652445 | 12.74808 | 8.346577 | 11.22613 | 6.491946 | 9.967403 | 23.08883 |
| 1 | 9 | GSM1688671_MG1999060203AA.CEL | 9.685405 | 6.554032 | 9.562136 | 13.67293 | 8.979562 | 12.31431 | 6.403698 | 11.93178 | 28.87696 |
| 1 | 14 | GSM1688672_MG1999060218AA.CEL | 9.449844 | 6.786945 | 9.684008 | 13.2002 | 9.375108 | 11.79982 | 6.757745 | 11.73179 | 24.04546 |
| 1 | 16 | GSM1688674_MG1999052809AA.CEL | 9.88539 | 6.883267 | 10.2145 | 12.09041 | 8.903015 | 11.3647 | 7.033846 | 10.38867 | 23.5504 |
| 1 | 18 | GSM1688675_MG2000030310AA.CEL | 9.217943 | 7.003656 | 10.0831 | 14.16435 | 8.759719 | 12.66944 | 6.232178 | 13.84452 | 27.23112 |
| 1 | 18 | GSM1688676_MG2000011809AA.CEL | 8.603184 | 6.589003 | 9.528941 | 14.08196 | 8.720932 | 13.16579 | 6.375811 | 13.23984 | 36.23194 |
| 1 | 19 | GSM1688678_MG1999052803AA.CEL | 9.597118 | 6.944865 | 10.25764 | 13.51642 | 8.925849 | 12.20201 | 6.161686 | 12.66601 | 24.86086 |
| 1 | 25 | GSM1688680_MG2000040501AA.CEL | 9.506997 | 7.072411 | 10.20773 | 14.44374 | 8.620747 | 13.94775 | 6.051061 | 13.50427 | 38.88328 |
| 1 | 26 | GSM1688681_MG1999052804AA.CEL | 9.699888 | 7.033007 | 10.13129 | 13.69873 | 8.943246 | 11.57387 | 6.99779 | 11.05214 | 21.18991 |
| 1 | 33 | GSM1688683_MG2000032807AA.CEL | 9.770228 | 7.808929 | 9.876607 | 14.19057 | 8.810408 | 12.8513 | 7.682666 | 12.5382 | 28.10794 |
| 1 | 38 | GSM1688685_MG2000040504AA.CEL | 9.233677 | 6.840031 | 10.20321 | 14.24974 | 9.037327 | 12.91292 | 6.486384 | 13.81122 | 30.2037 |
| 1 | 39 | GSM1688686_MG1999060216AA.CEL | 9.271299 | 6.649309 | 9.929915 | 14.10946 | 8.686552 | 12.71887 | 6.457615 | 13.13137 | 30.89089 |
| 1 | 39 | GSM1688688_MG2000040510AA.CEL | 9.512067 | 8.358007 | 10.16737 | 14.24215 | 8.719435 | 13.23902 | 6.243687 | 13.24867 | 26.5447 |
| 1 | 42 | GSM1688690_MG2000032011AA.CEL | 9.858217 | 7.456149 | 10.12651 | 14.32242 | 9.464067 | 12.4398 | 6.817233 | 13.12177 | 22.45307 |
| 1 | 65 | GSM1688692_MG2000031504AA.CEL | 9.193277 | 7.195384 | 10.36071 | 14.76284 | 8.512977 | 13.30115 | 6.29301 | 13.86386 | 31.76172 |
| 1 | 92 | GSM1688693_MG1999052819AA.CEL | 9.527874 | 7.43569 | 10.4186 | 12.32119 | 9.256426 | 11.32567 | 7.077995 | 9.968133 | 20.58872 |
| 1 | 102 | GSM1688695_MG1999060214AA.CEL | 10.14796 | 6.982138 | 10.19196 | 13.14391 | 9.178768 | 12.05346 | 6.76664 | 11.09605 | 25.82351 |
| 0 | 24 | GSM1688697_MG2000030309AA.CEL | 8.994142 | 7.997131 | 9.850502 | 13.58725 | 8.241682 | 13.58738 | 6.155552 | 13.604 | 33.71298 |
| 0 | 25 | GSM1688698_MG1999052802AA.CEL | 9.487323 | 6.682805 | 10.15752 | 13.87388 | 8.843002 | 11.97154 | 6.553101 | 12.69237 | 24.03065 |
| 0 | 27 | GSM1688700_MG1999052806AA.CEL | 10.07401 | 6.97876 | 11.21318 | 13.13668 | 8.82884 | 11.27193 | 6.607927 | 11.36812 | 17.99349 |
| 0 | 28 | GSM1688702_MG2000032802AA.CEL | 9.613485 | 7.378468 | 10.04656 | 14.76299 | 9.130386 | 13.33118 | 6.581051 | 13.16878 | 31.15301 |
| 0 | 33 | GSM1688703_MG2000011803AA.CEL | 9.537179 | 6.153006 | 10.02181 | 12.61414 | 8.802011 | 12.1478 | 6.608183 | 12.17594 | 31.42429 |
| 0 | 34 | GSM1688704_MG2000030702AA.CEL | 9.506706 | 8.379751 | 10.23963 | 13.81729 | 8.96658 | 12.05253 | 6.824351 | 11.06163 | 19.02473 |
| 0 | 35 | GSM1688705_MG1999052810AA.CEL | 9.446866 | 7.190975 | 10.01056 | 13.60496 | 9.636258 | 11.9731 | 6.955357 | 10.79729 | 24.1263 |
| 0 | 35 | GSM1688706_MG1999052816AA.CEL | 9.732129 | 7.479879 | 10.333 | 12.3273 | 9.170167 | 10.96314 | 6.472175 | 10.41685 | 15.25043 |
| 0 | 36 | GSM1688707_MG1999061601AA.CEL | 9.477144 | 6.496188 | 10.18767 | 13.02303 | 8.932785 | 10.56848 | 6.441276 | 11.31578 | 14.36201 |
| 0 | 39 | GSM1688708_MG2000011813AA.CEL | 9.819811 | 8.08144 | 10.37958 | 12.54224 | 9.069907 | 11.50481 | 7.102838 | 10.21061 | 18.40624 |
| 0 | 39 | GSM1688709_MG2000040513AA.CEL | 9.347557 | 7.125829 | 10.16966 | 14.37517 | 8.595604 | 12.41476 | 6.463076 | 13.34445 | 24.81612 |
| 0 | 41 | GSM1688710_MG2000040508AA.CEL | 9.310379 | 6.897359 | 10.23227 | 14.2007 | 8.617203 | 12.39687 | 6.338422 | 13.66397 | 25.46643 |
| 0 | 42 | GSM1688711_MG1999060210AA.CEL | 9.24931 | 7.219179 | 10.65086 | 14.05165 | 9.055404 | 12.03742 | 7.873942 | 11.9625 | 24.45989 |
| 0 | 45 | GSM1688712_MG1999060207AA.CEL | 9.637771 | 6.562022 | 10.10645 | 12.3183 | 8.507624 | 11.49438 | 7.142814 | 12.53905 | 24.12459 |
| 0 | 46 | GSM1688713_MG1999060206AA.CEL | 10.25473 | 7.701066 | 10.55031 | 13.69749 | 8.892034 | 12.56433 | 6.294168 | 12.14486 | 24.42267 |
| 0 | 51 | GSM1688714_MG1999062402AA.CEL | 9.833923 | 7.253139 | 10.15903 | 12.39623 | 9.097042 | 11.09736 | 6.720407 | 10.37933 | 17.95425 |
| 0 | 52 | GSM1688715_MG1999062401AA.CEL | 10.12411 | 7.475558 | 9.97659 | 12.02001 | 8.802403 | 11.32232 | 6.745489 | 10.27237 | 20.02991 |
| 0 | 53 | GSM1688716_MG1999060215AA.CEL | 9.580714 | 8.093503 | 10.11118 | 13.54802 | 9.113236 | 12.28188 | 6.642422 | 11.23393 | 22.38427 |
| 0 | 57 | GSM1688717_MG1999060702AA.CEL | 9.788375 | 7.123374 | 9.658293 | 13.21532 | 8.700211 | 12.05093 | 6.46099 | 11.98556 | 24.62221 |
| 0 | 60 | GSM1688718_MG1999052813AA.CEL | 9.823875 | 7.554253 | 10.20243 | 12.4084 | 9.400076 | 11.24625 | 7.895143 | 9.831709 | 19.87234 |
| 0 | 62 | GSM1688719_MG1999060213AA.CEL | 9.561469 | 7.50618 | 10.12277 | 13.03277 | 8.753587 | 12.17168 | 6.420496 | 12.13844 | 24.11178 |
| 0 | 64 | GSM1688720_MG1999052814AA.CEL | 9.358624 | 8.323537 | 10.20438 | 12.28863 | 9.089589 | 10.99511 | 6.342521 | 10.6076 | 11.70779 |
| 0 | 66 | GSM1688721_MG1999052820AA.CEL | 9.828383 | 6.949756 | 10.79056 | 12.63271 | 9.034198 | 11.46772 | 6.966752 | 11.72009 | 21.10495 |
| 0 | 68 | GSM1688722_MG1999060217AA.CEL | 9.091645 | 8.243123 | 9.854722 | 13.91607 | 8.914332 | 12.04204 | 7.02157 | 11.67695 | 19.54533 |
| 0 | 68 | GSM1688723_MG2000041102AA.CEL | 9.27608 | 7.145211 | 9.997538 | 14.07667 | 8.504193 | 13.25775 | 6.159036 | 13.27048 | 33.43469 |
| 0 | 70 | GSM1688724_MG1999052805AA.CEL | 9.567837 | 7.293688 | 10.58979 | 12.92072 | 9.204175 | 11.25832 | 7.0226 | 11.55624 | 17.29395 |
| 0 | 72 | GSM1688725_MG1999052801AA.CEL | 9.841695 | 7.448617 | 10.3519 | 12.58381 | 9.392021 | 11.50568 | 6.617169 | 10.51056 | 19.84806 |
| 0 | 74 | GSM1688726_MG1999052807AA2.CEL | 8.980738 | 6.549277 | 9.949073 | 12.93386 | 8.665168 | 10.9106 | 6.549618 | 12.07832 | 17.80194 |
| 0 | 79 | GSM1688727_MG1999052808AA.CEL | 9.580327 | 6.551835 | 9.958839 | 13.46301 | 9.162238 | 11.20502 | 6.680553 | 11.38101 | 19.34581 |
| 0 | 79 | GSM1688728_MG2000031506AA.CEL | 9.168399 | 8.477666 | 10.85239 | 13.90497 | 8.764436 | 11.01275 | 8.187035 | 12.8913 | 8.252021 |
| 0 | 80 | GSM1688729_MG2000041202AA.CEL | 9.098755 | 7.056347 | 9.925116 | 14.08559 | 8.628965 | 12.67339 | 6.295103 | 13.29529 | 28.36314 |
| 0 | 84 | GSM1688730_MG1999060211AA.CEL | 9.405941 | 6.575534 | 9.824975 | 13.59246 | 9.100279 | 12.48788 | 6.494546 | 12.80852 | 29.67813 |
| 0 | 85 | GSM1688731_MG1999060212AA.CEL | 9.93681 | 7.072507 | 9.779027 | 14.28904 | 8.53132 | 12.22455 | 7.453548 | 12.12654 | 25.84006 |
| 0 | 87 | GSM1688732_MG1999060201AA.CEL | 9.379318 | 7.195467 | 10.79341 | 13.19743 | 8.814393 | 12.01315 | 6.904946 | 11.56297 | 25.22282 |
| 0 | 87 | GSM1688733_MG1999052817AA.CEL | 9.344547 | 6.526678 | 10.22373 | 13.78031 | 8.738272 | 12.57768 | 6.258543 | 12.74065 | 30.72082 |
| 0 | 97 | GSM1688734_MG2000040505AA.CEL | 9.259887 | 7.160379 | 10.04632 | 14.62886 | 8.890927 | 13.32629 | 6.155556 | 13.94908 | 31.55618 |
| 0 | 97 | GSM1688735_MG2000041207AA.CEL | 9.241801 | 7.917021 | 10.15433 | 14.01695 | 8.486709 | 13.10875 | 7.69544 | 13.01236 | 30.86519 |
| 0 | 108 | GSM1688736_MG1999060209AA.CEL | 9.877347 | 6.370322 | 9.937656 | 13.04827 | 8.560742 | 12.29392 | 6.450614 | 11.69243 | 31.37041 |
| 0 | 108 | GSM1688737_MG2000041203AA.CEL | 10.40599 | 8.090435 | 9.938493 | 13.01905 | 8.615333 | 11.73478 | 6.75816 | 11.53142 | 17.48467 |
| 0 | 130 | GSM1688738_MG1999060902AA.CEL | 9.775606 | 7.100456 | 10.11728 | 11.74823 | 8.692725 | 10.68018 | 6.421132 | 10.25116 | 16.22199 |
| 0 | 24 | GSM1688739_MG1999062404AA.CEL | 9.651484 | 7.595294 | 9.926548 | 12.9506 | 8.730961 | 11.27971 | 6.495726 | 11.54918 | 15.97987 |

**Supplementary Table 3.** The gene level of the Asian cohort together with the gene-signature score and Translated Score.

| ID | Score | Patient Strata | ASIC2 | CHRM3 | CHRM4 | EEF1A1 | GPR162 | HSP90AB1 | PTPRR | RPS23 | Translated Score |
| --- | --- | --- | --- | --- | --- | --- | --- | --- | --- | --- | --- |
| T2056 | 2.780574219 | High risk | 6.542913 | 11.56261 | 6.711784 | 20.75109 | 7.990958 | 17.08413 | 9.077894 | 17.18195955 | 40.94716883 |
| T1095 | 2.722242246 | High risk | 7.972482 | 8.405874 | 5.601722 | 20.56698 | 9.884307 | 17.29898 | 6.859656 | 18.14355047 | 50.04556111 |
| T3698 | 1.805842803 | High risk | 6.836892 | 10.18763 | 6.425927 | 19.50619 | 9.045068 | 16.24929 | 7.268364 | 17.4669834 | 37.00139999 |
| T1425 | 1.254466284 | High risk | 5.485068 | 6.765468 | 5.754783 | 20.3566 | 9.538416 | 16.39633 | 5.87459 | 16.97740294 | 53.42358717 |
| T739 | 0.693576168 | High risk | 6.039797 | 7.862756 | 5.776771 | 19.58921 | 9.391025 | 15.84594 | 6.395525 | 16.17520859 | 45.41630078 |
| T1090 | 0.655460388 | High risk | 5.738339 | 7.774661 | 6.415373 | 19.63615 | 10.04825 | 15.52142 | 7.00789 | 16.23103709 | 42.66211411 |
| T579 | 0.223141274 | High risk | 7.035453 | 8.005914 | 5.608875 | 18.71401 | 6.679606 | 15.46545 | 5.745416 | 14.77809648 | 46.25169083 |
| TM28 | 0.122541667 | High risk | 6.048544 | 8.570335 | 6.691085 | 19.14692 | 10.94836 | 15.13667 | 7.767435 | 15.57682296 | 36.11214864 |
| CH53 | 0.037060383 | High risk | 5.912753 | 6.54959 | 6.422724 | 19.91454 | 10.0315 | 15.87631 | 6.24456 | 16.87651344 | 49.44478886 |
| T1950 | -0.023814636 | High risk | 6.767157 | 6.946242 | 5.74124 | 19.83396 | 10.15828 | 16.29562 | 6.092183 | 16.60091233 | 50.80065217 |
| RT1294 | -0.324457092 | High risk | 7.788748 | 11.53658 | 7.551922 | 19.30553 | 9.557871 | 15.69809 | 11.67658 | 15.18304312 | 32.46785468 |
| T692 | -1.02309777 | High risk | 8.583464 | 6.590207 | 5.534298 | 19.3998 | 8.464312 | 16.27668 | 6.692753 | 15.92688525 | 55.06810976 |
| T3256 | -1.297844628 | High risk | 7.323128 | 5.99968 | 5.812472 | 20.08747 | 9.530078 | 16.42929 | 7.951153 | 16.62728916 | 59.00436614 |
| RT841 | -1.3253373 | High risk | 6.390357 | 10.99658 | 7.387343 | 18.63136 | 10.83124 | 15.40742 | 9.339481 | 15.04839601 | 30.37292384 |
| T113 | -1.653156733 | High risk | 5.287988 | 6.404889 | 5.703429 | 19.15985 | 11.57416 | 15.62643 | 5.880683 | 15.44930214 | 49.23576261 |
| T709 | -2.021946387 | High risk | 7.573071 | 9.849233 | 6.777391 | 18.50797 | 8.956816 | 15.33099 | 9.297299 | 14.42532983 | 37.55624933 |
| T4394 | -2.223430122 | High risk | 10.26068 | 12.99976 | 12.31688 | 18.45761 | 11.26977 | 14.94363 | 12.44435 | 16.14637128 | 12.57963217 |
| T4060 | -2.46890731 | Average risk | 7.730298 | 10.68586 | 7.135328 | 18.17501 | 11.58901 | 15.33237 | 9.519262 | 14.73365316 | 29.91861197 |
| RT593 | -2.607697693 | Average risk | 9.472492 | 12.08637 | 10.26776 | 18.49043 | 9.678334 | 15.03164 | 11.42365 | 15.09980933 | 21.57967188 |
| T1444 | -2.642950501 | Average risk | 5.84789 | 12.49273 | 6.413781 | 18.2756 | 10.70749 | 15.03384 | 9.709076 | 14.34439401 | 22.6496467 |
| TM111 | -2.669383609 | Average risk | 5.939179 | 11.42112 | 7.32112 | 18.33191 | 11.27906 | 14.72753 | 9.452879 | 14.1879116 | 23.47536921 |
| TM4 | -2.757154366 | Average risk | 9.74306 | 12.89748 | 10.45298 | 18.04818 | 10.19875 | 14.65791 | 10.77384 | 14.8712286 | 13.0454342 |
| CH107 | -2.863168973 | Average risk | 9.648995 | 10.5403 | 9.841843 | 18.23943 | 10.42801 | 15.22571 | 10.08096 | 14.86441163 | 28.68701777 |
| T208 | -3.145652667 | Average risk | 6.61273 | 6.623203 | 5.669503 | 18.90306 | 11.32816 | 15.19325 | 5.468832 | 14.84249673 | 43.32716681 |
| T2792 | -3.218390078 | Average risk | 8.611886 | 11.25559 | 6.854203 | 19.33962 | 10.14125 | 16.01486 | 7.905818 | 15.88713307 | 28.90351148 |
| RT1335 | -3.378681189 | Average risk | 7.999667 | 10.26572 | 5.794881 | 17.91023 | 9.936115 | 15.48712 | 6.02007 | 13.98099337 | 32.41900225 |
| T1015 | -3.400940455 | Average risk | 8.878698 | 11.25841 | 7.098763 | 19.12224 | 9.79858 | 15.59246 | 11.01387 | 15.01827677 | 30.78686661 |
| T4201 | -3.455883712 | Average risk | 7.266679 | 12.28703 | 7.12864 | 18.55203 | 10.12275 | 15.51069 | 9.020891 | 14.81185414 | 25.08185429 |
| CH89 | -3.500750976 | Average risk | 5.742602 | 13.09905 | 7.993185 | 17.44867 | 11.47336 | 14.61903 | 10.625 | 13.9281466 | 17.66234718 |
| T1184 | -3.791195472 | Average risk | 8.367868 | 9.133591 | 6.086426 | 19.26216 | 11.20962 | 16.01174 | 7.070706 | 15.82898251 | 37.19938685 |
| T4325 | -3.878230357 | Average risk | 5.899473 | 12.96395 | 6.285002 | 17.54612 | 10.9181 | 14.98785 | 9.619386 | 14.00803895 | 21.22200274 |
| T2044 | -3.889206573 | Average risk | 6.927896 | 12.93224 | 7.287453 | 17.55802 | 11.4599 | 14.89226 | 10.06237 | 13.83001601 | 18.96079596 |
| T4053 | -3.907145212 | Average risk | 10.23022 | 8.615587 | 9.58079 | 17.87507 | 11.11093 | 15.53032 | 10.79892 | 14.05627145 | 42.12795577 |
| T4247 | -4.204024016 | Average risk | 8.52525 | 10.44625 | 5.490589 | 18.6292 | 10.95752 | 15.50063 | 7.936899 | 14.98873975 | 29.61717207 |
| T4046 | -4.338725503 | Average risk | 6.694831 | 12.32424 | 6.752434 | 18.10872 | 10.8206 | 15.19752 | 9.461377 | 14.57851921 | 23.38912606 |
| T4436 | -4.372251778 | Average risk | 6.736118 | 7.932264 | 5.973856 | 17.9937 | 11.64435 | 14.7048 | 6.731907 | 14.30184876 | 35.65458691 |
| T4346 | -4.379428972 | Average risk | 7.916892 | 11.94705 | 8.504365 | 17.86075 | 11.36134 | 14.5861 | 8.271895 | 13.76086333 | 16.40302319 |
| T3635 | -4.466273673 | Average risk | 8.498768 | 11.56675 | 6.287608 | 18.93102 | 9.646715 | 15.36634 | 11.6313 | 14.76039969 | 29.52798813 |
| T1307 | -4.596569665 | Average risk | 7.62507 | 13.20848 | 8.273833 | 18.1375 | 11.84833 | 15.27427 | 12.27935 | 14.4119582 | 20.93917475 |
| TM52 | -4.606059968 | Average risk | 7.177836 | 12.70583 | 8.170949 | 16.57784 | 11.65007 | 14.58468 | 10.41573 | 13.47555898 | 19.01970509 |
| T4042 | -4.640642685 | Average risk | 5.964698 | 12.72188 | 7.53609 | 17.9415 | 10.8071 | 14.97239 | 9.324668 | 14.40671403 | 20.36794748 |
| T66 | -4.690616858 | Average risk | 7.689756 | 6.744139 | 5.749206 | 17.82141 | 11.1145 | 14.32886 | 6.039725 | 14.32078128 | 36.68595569 |
| RT742 | -4.921974375 | Average risk | 7.135126 | 12.88009 | 8.315321 | 17.69492 | 11.17455 | 15.20041 | 12.88623 | 13.86900954 | 25.79125411 |
| T1882 | -5.15721891 | Average risk | 5.852656 | 12.50171 | 7.874831 | 17.53944 | 11.46755 | 14.80076 | 9.944421 | 13.60658614 | 21.55641782 |
| T1686 | -5.35972032 | Average risk | 9.08458 | 12.28691 | 7.582504 | 17.39435 | 11.38779 | 14.62405 | 10.98011 | 13.71974028 | 18.50531007 |
| T1080 | -5.359750148 | Average risk | 10.03929 | 9.665118 | 5.830799 | 18.84583 | 11.54358 | 15.34678 | 8.17007 | 15.23082619 | 28.67167414 |
| T3654 | -5.491731087 | Average risk | 8.329499 | 10.32709 | 7.015766 | 18.0834 | 11.57317 | 14.84033 | 10.11655 | 14.55027468 | 27.39805825 |
| CH127 | -5.927499679 | Average risk | 8.906554 | 9.834868 | 5.893285 | 19.06036 | 11.53422 | 15.28115 | 7.866179 | 15.82493147 | 27.02719721 |
| CH1 | -6.210945073 | Average risk | 8.300615 | 13.05335 | 7.925342 | 18.50239 | 10.8418 | 14.67814 | 12.56662 | 14.33938159 | 16.38014292 |
| T1180 | -6.806594687 | Average risk | 9.015351 | 10.99448 | 7.345792 | 17.86917 | 11.04462 | 14.51857 | 11.1648 | 13.9860569 | 23.43993037 |
| T4186 | -8.984518371 | Average risk | 10.06401 | 12.66627 | 10.38186 | 18.93326 | 10.09032 | 14.95546 | 11.04819 | 15.75800997 | 14.41815198 |
| T4356 | -10.98797539 | Average risk | 11.14686 | 11.63682 | 11.54192 | 18.18235 | 11.27254 | 14.85697 | 10.56447 | 14.76813182 | 17.14224368 |
